# Analysis of the survival of agricultural exporting firms in Peru, 2009-2019

**DOI:** 10.12688/f1000research.158554.1

**Published:** 2024-11-27

**Authors:** Rogger Orlando Morán Santamaría, Yefferson Llonto Caicedo, Dante Godofredo Supo Rojas, Willy Darwin Llatas Díaz, Martin Hamilton Wilson Huamanchumo, Ofrmar Dionell Jiménez Garay, José Alberto Uribe Del Aguila, Pietro Pablo Guissepi Dondero Cassano, Percy Junior Castro Mejía

**Affiliations:** 1 La Libertad, Universidad Cesar Vallejo, Trujillo, La Libertad, Peru; 2 Lambayeque, Universidad Nacional Pedro Ruiz Gallo, Lambayeque, Lambayeque, Peru; 3 Universidad de San Martín de Porres, Universidad de San Martin de Porres Facultad de Medicina Humana, La Molina, Lima Region, Peru; 4 Cajamarca, Universidad Nacional de Cajamarca, Cajamarca, Cajamarca, Peru; 5 Ica, Universidad Nacional San Luis Gonzaga de Ica, Ica, Ica, Peru; 6 Lima, Universidad Norbert Wiener, Lima District, Lima Region, Peru

**Keywords:** Agricultural exporting firms, Export resilience, Economic sustainability, Peru's agricultural exports, Business survival analysis

## Abstract

**Background:**

At the international level, the survival of exporting companies represents a critical issue in a context of heightened uncertainty and intensified competition within the framework of the country’s commercial opening. This is a context in which different companies are born and die as a result of the interaction between the market and other factors. The objective of this research was to analysis of the survival of exporting agricultural companies in Peru, 2009-2019. To this end, data from the Commission for the Promotion of Peru for Exports and Tourism (Promperu) was utilised.

**Methods:**

The methodological contribution of the research is based on the quantitative approach, of basic type with a descriptive elk; being the population that involves a total of data of the agricultural exporting companies of Peru in the period 2009-2019 and the sample is census and the non-parametric statistical technique used was the Kaplan Meier estimate for the estimation of the survival rate.

**Results:**

Exports of Peru’s non-traditional agricultural sector in FOB value have had an average annual growth of 12% in terms of FOB value and 9% in terms of volume exported; the entry rate of new agro-exporting agricultural companies reached an average growth of 2.3% and the exit rate reached an average of 2.2% in the period 2009-2019.

**Conclusions:**

The survival of exporting companies in the non-traditional agricultural sector is critical, where 89% of them survive only one year, while in the second year only 75% survive and in the sixth year only 33% survive.

## 1. Introduction

In their relentless pursuit of competitiveness, expansion and growth, companies increasingly rely on exports as a pivotal strategy to attain their desired outcomes. In this context, economic integration has emerged as a pivotal conduit for facilitating firms’ access to the export market ( Teye Gaga et al., 2023). Empirical studies have demonstrated the significance of economic integration in determining export survival rates. Martuscelli and Varela (2018) posit that economic integration enhances the likelihood of success in trade relations by reducing the costs associated with policies, providing strategic information about destination markets and reducing competition, thereby significantly increasing the probability of survival in export markets. Furthermore, from a policy perspective, it is of paramount importance to comprehend the obstacles that developing countries encounter in their endeavours to diversify and sustain their export survival. Their economic growth is contingent upon their capacity to produce and trade goods in a competitive manner within the global marketplace ( Kim et al., 2024).

The rate of business failure has increased markedly since the onset of the SARS-CoV-2 pandemic, rising from 4.5% to 12.1% by 2020 ( Gupta et al., 2024; Gyarmati, 2024; Lin et al., 2024). In light of the aforementioned factors, the survival of exporting firms represents a critical issue in the context of globalisation and increasing international competition. As economies become more interconnected, firms that rely on international markets face unique challenges that affect their sustainability and growth. The extant literature suggests that the survival of these firms is influenced by a variety of factors, including innovation, resource management and adaptation to global markets. The aforementioned conditions ( Cabaleiro & Mendi, 2024; Almeida Lucero, 2024; Derindag et al., 2024; Jorge-Martín & Fernández, 2024; Mercan et al., 2024).

The contribution of the agro-export sector to the global economy is significant, encompassing not only the generation of employment but also the production of food aligned with the fight against food insecurity. However, in recent years, it has demonstrated a heightened vulnerability in its capacity to compete in international markets, necessitating survival at the global level. This is largely attributed to the numerous barriers it faces in accessing the international market, particularly in a context of heightened complexity and an evolving competitive landscape ( Casillas et al., 2020; Guimarães et al., 2021; Atici, 2024; Montes Ninaquispe, et al., 2024a; Rodríguez Abraham et al., 2024).

It is crucial to comprehend these elements in order to devise strategies that not only enable exporting enterprises to endure but also to flourish in an ever-evolving environment. Peruvian agro-exporting companies have discerned that there are both intrinsic and extrinsic factors impeding the expansion of businesses in international markets. The internal factors pertain to managerial focus and commitment, knowledge and experience, as well as functional and marketing information. In contrast, the external factors relate to the destination and origin market ( Malca & Donet, 2015). It is therefore important to identify the survival of exporting companies in Peru during the period 2009-2019.

In the conceptual contribution, exporting is defined as an economic activity in which countries sell products or services to the international market. This is done in the context of having competitive advantages that contribute to the enrichment of countries and generate more international reserves, which in turn can be used to improve the employment of the inhabitants ( Safari & Saleh, 2020).

Nevertheless, an investigation into the factors impeding the establishment and sustainability of export enterprises has revealed that the primary explanation can be attributed to the formidable international competition, coupled with the presence of weak procedures and challenges in identifying new international markets ( Leonidou, 1995).

In their seminal work, Bilkey and Tesar (1977) proposed a six-stage model to explain the exporting process of firms. They identified four key types of export barriers: attitudinal, structural, operational, and others. These barriers prevent firms from initiating export activities. As one progresses through the stages of exporting, it becomes evident that the complexity of understanding business practices in foreign markets intensifies, largely due to the proliferation of intricate specifications and standards in international markets ( Alexandrides, 1971; Leonidou, 1995).

In this context, Ando et al. (2022) argue that an international presence is essential for an exporting company to be sustainable in the emerging countries of China, India and Brazil, which compete with the main developed countries of North America and Europe. Over time, the attitude of a company must evolve from a mere presence in new markets to a more structural contribution that allows it to expand. This requires clear and precise strategies to conquer new markets, as well as the relevant resources for companies to remain in the market and withstand the pressures exerted on both prices and profit margins.

In Latin America, the primary challenges faced by exporting companies are the lack of investment and the restricted capacity to export in an efficient, effective, and profitable manner. This, coupled with the limited utilisation of technology, impedes innovation and the development of new product lines, resulting in a lack of competencies that are essential for the long-term sustainability of companies ( Zhang et al., 2017).

In their research, Casillas et al. (2020) identify the contributions of the international arena based on the age of entry and export experience, which influence the initial stage of the internationalisation process. They consider age of entry to be a significant factor in explaining the volume of export intensity and in the gradual process of further market entry. This reveals its greater importance to the internationalisation strategy.

In the case of Peru, Chávez (2022) posits that the primary factors influencing the survival of small firms in the agro-export sector are portfolio diversification, the experience of the exporter, and the effort to have a larger exportable supply. These factors demonstrate that exogenous shocks play a pivotal role in determining the probability of a firm succeeding in the export market.

Conversely, Pantaleón et al. (2021) posit that the issue facing avocado exporting companies is that their survival rate is declining. This is due to three causal factors related to product and market diversification, market heterogeneity, and information networks. These factors are crucial aspects of the dynamics of Peruvian exporting business growth. Additionally, there are structural factors that contribute to the decline in survival rates. These are the complexity of the environment in which the companies operate and the characteristics of the businessmen themselves.

In the view of Carrasco et al. (2023) the ability of agro-exporting companies to survive in the context of a lack of access to sources of financing and technology is contingent upon their capacity to adapt to an increasingly challenging environment. The probability of survival is reduced, and the likelihood of failure is heightened, due to the crucial role of technology in enhancing production volumes.

To date, there have been few studies that have attempted to identify the factors that contribute to the survival of exporting companies. These studies have found that the barriers to exporting involve both internal and external factors, including the global context of the international market itself and the company’s internal environment. This demonstrates that the knowledge gap pertains to the particular impact of acquired strategies on the survival of exporting firms in emerging markets. This, in turn, constrains the comprehension of how these practices can enhance business sustainability in these contexts. Conversely, there is a paucity of research examining the impact of strategic international expansion decisions on the long-term sustainability of exporting firms in highly competitive industries. This perpetuates an incomplete understanding of optimal practices in such contexts.

The extant literature indicates that innovation and the adoption of sustainable practices are pivotal determinants of the survival of exporting firms. Firms that integrate sustainability strategies into their business model tend to demonstrate superior performance in terms of innovation and labour productivity, which in turn translates into enhanced survival rates ( Cabaleiro & Mendi, 2024; Montes Ninaquispe et al., 2024b). Furthermore, recent studies have indicated that companies that implement corporate social responsibility (CSR) practices not only enhance their reputation but also gain a competitive advantage in international markets ( Esteve et al., 2020). Nevertheless, despite these findings, there are still limitations in the research, such as the lack of longitudinal studies that analyse the long-term impact of these practices on business survival.

The problem of the survival of exporting companies becomes even more evident in the current context, where economic crises and changes in trade policies can have a devastating impact. The principal objective of this study was to carry out an analysis of the survival of exporting agricultural companies in Peru, 2009-2019. The specific aims were to describe the entry and exit of exporting agricultural companies in Peru from the international market, to describe the survival rate of exporting agricultural companies in Peru, and to carry out a comparative analysis of the survival time of exporting agricultural companies in Peru.

The rationale for this study is the necessity for a more profound comprehension of the evolution of the survival of agro-export companies in Peru, and the significance of the agro-export sector in the economic sphere, particularly in terms of employment generation and its substantial impact on the national economy. Given its role as a key driver of the Peruvian economy, it is vital to devise effective strategies to enhance competitiveness and contribute to long-term growth. This is particularly important given the substantial volume of exports, which contribute to increased foreign currency income and job creation. Conversely, it is of the utmost importance to develop efficacious and efficient public policies related to foreign trade, with the objective of effectively managing export companies and ensuring their long-term sustainability.

## Literature review

The recent empirical evidence presented by Chang et al. (2022) illustrates the challenges faced by small and medium-sized exporting firms in developing countries with regard to the management of product and process innovation. Consequently, the survival of exporting firms is contingent upon the manner in which they innovate their products for export.

In a similar vein, Leurcharusmee et al. (2022) in Thailand have identified that the survival of MSEs is contingent upon a number of factors, including location, number of employees and business model fit, as well as the ability to operate with social distancing and utilise online marketing. Their findings suggest that the relevant factors influencing the survival probability trajectories of MSEs are those related to commercial factors, such as location, total assets, type of business and commercial operation.

Yllescas et al. (2021) highlight that Peru, Chile and Colombia have implemented diverse strategies to diversify their export supply, leading to a diversified strategic supply. This involves adopting production technology that is less vulnerable to changes in the international market and improving foreign trade policy to facilitate export growth.

In a study by Reyes (2021) the Logit methodology was used to identify the internal factors that determine the probability of an exporting company in the agricultural sector being sustainable. These factors included access to credit, product diversification and the experience of the entrepreneurs.

In this context Safari and Saleh (2020) posit that in emerging countries, internal and external factors have been identified with respect to innovation strategy, export marketing and business strategy. These factors pertain to the improvement of the export process and the motivation of companies to export, which may be achieved by the application of effective standards, the simplification of processes, the increase of incentives and other strategic initiatives, with the objective of facilitating export to regional and global markets.

In the study by Casillas et al. (2020) the results indicated the significance of market entry and its short-term impact. The age of entry was identified as a crucial factor in explaining the volume and intensity of exports. Exporting companies undergo a gradual process of acquiring the capabilities necessary for internationalisation. The relevance of the change in acquired capabilities at the beginning of exporting was also highlighted. It was observed that companies that export in their initial phase tend to have more experience than those that export in their late stages.

In contrast, while Esteve et al. (2020) found that the survival rate of new exporting manufacturing companies in Spain is higher than that of export relationships defined at the provincial, product, and destination levels. This positive effect is evidenced by the concentration of regular exporters at the provincial, product, and destination levels. Notably, the survival effectiveness of exporting companies is 75% for those whose exports to the same product exceed 10. This highlights the importance of orienting nearby companies to develop similar export activities through export promotion policies.

The analysis conducted by Pantaleón et al. (2021) revealed that, in the Peruvian context, avocado exporting companies achieved an entry rate of 33% in 2013 and 34% by 2020. Similarly, the exit rate reached 32% in 2013 and 29% by 2020. Additionally, the findings indicated that the survival factor is becoming increasingly challenging to achieve, particularly given the diminishing probability of survival over time. Notably, the survival factor was identified as the primary determinant of sustained enterprise, with 52% of companies surviving in the second year and a mere 6% surviving in the ninth year.

## Theoretical basis

### International trade

Theories of international trade have evolved over the centuries, offering a variety of explanations for the reasons behind and benefits of the exchange of goods and services between countries. These theories are fundamental to understanding the global economic dynamics and trade policies that countries adopt. The conventional trade theory (CTT) posits that trade originates from differences in technology or preferences. It asserts that trade begins with the identification of comparative advantages, which contribute to the creation of employment and economic growth. These factors are essential indicators of the commercial exchange between countries ( Fassio, 2018; Erdogan, 2014; Jones, 2010; Krishna, 2005; Leamer & Levinsohn, 1995).

From an economic perspective, exports serve as a catalyst for growth, as they contribute to the generation of value through the trade balance, thereby facilitating the generation of foreign currency and contributing to the gross domestic product. Furthermore, they play a pivotal role in diversifying the economy, enabling it to maintain competitiveness and adaptability in the face of global market dynamics. For exporting companies, the ability to navigate these challenges is crucial for their survival and long-term success ( De Gregorio, 2012; Gokmenoglu et al., 2015; Lim & Ho, 2013; Mahadevan & Suardi, 2008; Sunde, 2017; Yaghmaian, 1994).

### Business survival

In examining the strategies employed by exporting companies to ensure their survival in a dynamic business environment, it becomes evident that these firms utilise a range of strategies that facilitate their adaptation to the ever-evolving global market ( Vásquez et al., 2017). In this way, the company’s capacity to overcome export barriers is enhanced by constant innovation and a willingness to venture beyond the comfort zone where companies have validity in the market. This approach enables differentiation that improves global market positioning and increases the capacity of other companies in the business area, achieving improved profitability and reduced market risks. This, in turn, contributes to enhancing competitors’ abilities in the global market ( Deng et al., 2014; Ferragina & Mazzotta, 2014; Kao & Liu, 2022; Mas-Verdú et al., 2015; Pittiglio, 2023).

### Kaplan-Meier estimator

The contribution of the Kaplan-Meier method is a method of calculating survival over time that is conditional on a predictor. It has become one of the most widely used aspects of survival analysis studies, given that it allows for the calculation of the probability of survival in a given period. As a non-parametric estimator, it is based on the characteristics of the event of interest and the period of study, which are clearly defined. It provides an estimate of the probability of survival of businesses and censored observations in the probability of survival. The estimator is calculated as the fraction of observations in the relevant system that started from a certain amount of time ( Gémar et al., 2016).

The Kaplan-Meier survival analysis provides insight into the construction of a survival curve, which represents the probability that an exporting company will continue to operate in the market and achieve timely development towards the economies of the global market over a specified period. This is achieved by demonstrating that the survival curve can be constructed using information from a defined period to conduct a survival analysis. The global market is defined as the period from the commencement of exporting activities for a company until the cut-off date for calculating survival rates. This demonstrates that survival analysis necessitates an understanding of firm mortality as an event and the probability of its occurrence, employing statistical methodology ( Leurcharusmee et al., 2022).

## 4. Methods

### 4.1 Methodological design

***Type of research***

The methodological contribution of the research is based on the quantitative approach, of a basic type with a descriptive approach, given that use is made of numbers and mathematical operations that infer in the analysis of the companies for their survival, considering quantitative methods and techniques.

***Research design***

The analysed variables were not modified, as the research was conducted using a non-experimental design; this allowed for observing and analyzing the behavior of agricultural export companies within a specific context Peru during the period 2009-2019 without any intervention in the study conditions ( Hernández et al., 2010).

***Population***

The population involves a total of data from agricultural exporting companies in Peru in the period 2009-2019. The sample is equivalent to a census, considering that the entire population is involved in the research, as there are no exclusion criteria for access to information.

***Unit of analysis***

The unit of analysis involves the agricultural exporting company operating in Peru, covering the period 2009-2019 ( Infotrade, 2024).

***Data collection techniques and instruments***

Documentary analysis was used as a technique to measure the variables, and the observation guide, which is a tool used to collect data in a systematic and structured way during an observation or study, was used as an instrument.

***Data collection procedures***

The data analysed was obtained from the Commission for the Promotion of Peru for Exports and Tourism (Promperu), which is a public body attached to the Ministry of Foreign Trade and Tourism, compiling complete and accurate data to produce a complete and coherent analysis of the survival of agricultural exporting companies in Peru.

For the survival analysis, use is made of the Kaplan Meier estimator for the construction of the survival curve of the agricultural exporting companies, as well as the market entry and exit rate, whose non-parametric test is relevant for the analysis in the estimated time period from 2009 to 2019.

### 4.2 Data analysis

The analysis of the data provided by the Commission for the Promotion of Peru for Exports and Tourism (Promperu) in the period 2009-2019 starts by using the non-parametric statistical technique of Kaplan Meier estimator for the construction of the survival curve of agricultural exporting firms. Data analysis was carried out using the free software IBM SPSS Statistics 27 ( IBM, 2024).

The contribution of the Kaplan-Meier method involves a way of calculating survival over time that is conditional on a predictor and has become one of the most widely used aspects of survival analysis studies, considering that the probability of survival over a given period.

Emphasising the importance of determining the survival of exporting companies, which leads to the restructuring of the productive matrix, which in turn is explained by internal and external factors; as well as the understanding of the current situation of agricultural companies involved in exports and the capacity to remain in force over time ( Malca & Donet, 2015; Pantaleón et al., 2021).

Thus, the Kaplan Meier formula for the construction of the survival curve of agricultural exporting firms is empirically supported as follows considering *t* ≥ 0 ( Gonzáles, 2016):

$$\hat{S}\;\left(t\right)=\frac{\text{Number of observations}\geq t}{n}$$

In this context, a step function is represented that decreases by l/ *n* immediately after each observation of the lifetime of an individual or firm, provided that all observations are distinct. More generally, if there are *d* lifetimes equal to *t*, the empirical survival function (ESF) decreases by an amount *d*/ *n* immediately after *t*.

The estimator of the product limit, *S* ( *t*), is defined as:

$$P\;\left(T>t\right)=\hat{S}\;\left(t\right)=\Pi \frac{\mathrm{nj}-\mathrm{dj}}{\mathrm{nj}}$$

## 5. Results

The results show that exports of the non-traditional agricultural sector of Peru in FOB value had the highest increase in the years 2010 and 2011 with 29% and 42% respectively considering the previous year; while it faced its biggest fall in 2012 of -8%, with an average annual growth in the years of study of 12% in the period 2009-2019 as observed in Table 1.

**Table 1.** Main indicators of Peru's non-traditional agricultural sector exports, 2009-2019.

Indicator	FOB value Thousands USD	Weight TM	Unit value	Companies
2009	2,464,133	1,663,563	1.48	4,961
2010	3,177,680	1,946,898	1.63	4,959
2011	4,519,345	2,218,458	2.04	4,959
2012	4,153,775	2,249,491	1.85	4,962
2013	4,197,164	2,365,440	1.77	4,966
2014	5,065,861	2,700,313	1.88	4,971
2015	5,113,235	2,716,189	1.88	4,968
2016	5,565,302	3,013,313	1.85	4,969
2017	5,930,440	3,197,363	1.85	4,974
2018	6,619,578	3,658,954	1.81	4,984
2019	7,073,705	3,904,937	1.81	4,984

*Note.* Obtained from own elaboration of the Promperu database, 2009-2019.

On the other hand, in terms of exports expressed in metric tonnes (MT), the highest growth was in the period 2010, 2018 and 2014 with a growth of 17%, 14% and 14% respectively; while the lowest growths were reflected in the period 2015 and 2012 which reached a growth of 0.6% and 1.4% respectively, considering an average annual growth in the period 2009 to 2019 of 9% in the exported volume expressed in tonnes.

**Figure 1.**
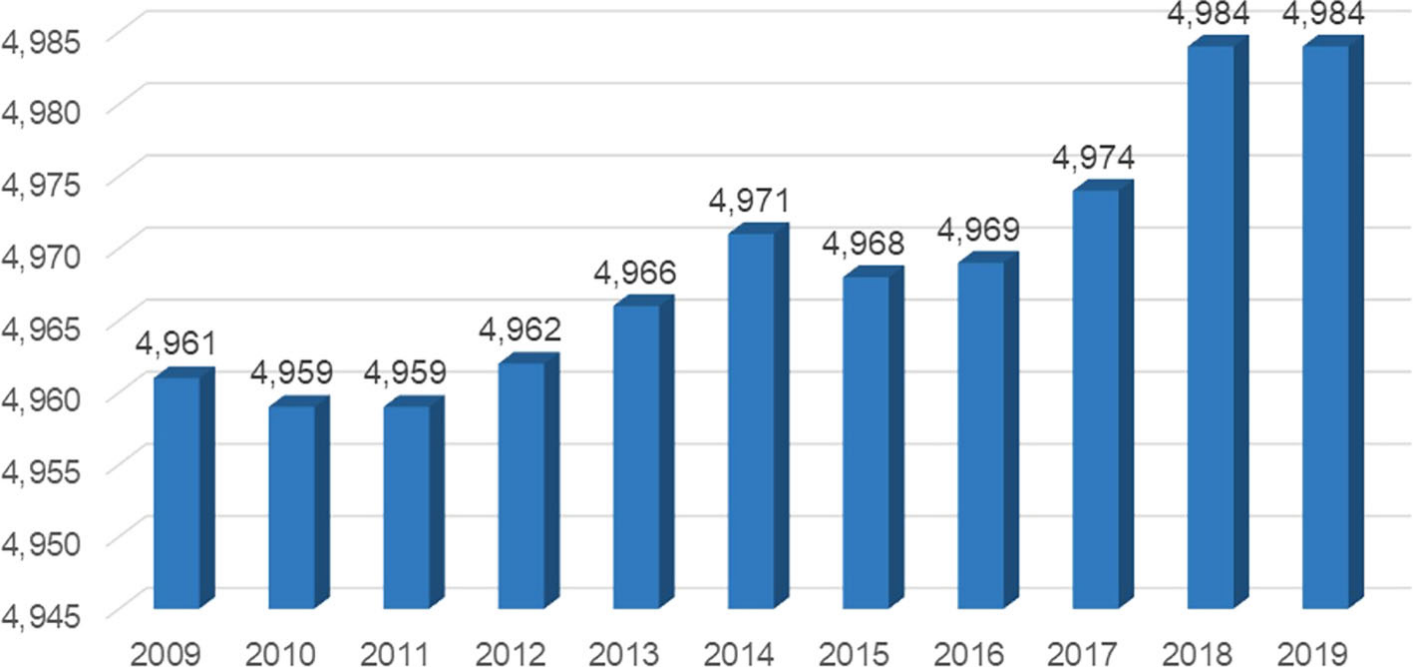
Net balance of exporting firms in Peru's non-traditional agricultural sector, 2009-2019. *Note.* Obtained from own elaboration of the Promperu database, 2009-2019.

On the other hand, with regard to the number of companies where a growing trend was evidenced from the year 2015 which reached 4968 companies in the non-traditional agricultural sector that carried out exports, and by 2019 reached 4984 companies in the sector under study as observed in Figure 1.

In Table 2, it was observed that the highest inflows of new agricultural enterprises expressed in the rate of inflow dedicated to export were found in the years 2012 with a growth of 2.6%; followed by the year 2014 and 2018 which had the same growth rate; while in the years 2015 reached the rate of 3.1% and for the year 2019 reached the rate of 3.5%; having an average growth rate of 2.3%; while the outflow rate were higher in the years 2012 and 2014 with a rate of 2.5%, in the year 2015 which reached a rate of 3.2% and in the year 2019 which reached a rate of 3.5%, having on average an outflow rate of 2.2%.

**Table 2.** Market entry and exit rate of Peru's non-traditional agricultural sector. 2009-2019.

Period	2009	2010	2011	2012	2013	2014	2015	2016	2017	2018	2019
Total		4,959	4,959	4,962	4,966	4,971	4,968	4,969	4,974	4,984	4,984
Entrance		98	84	128	54	127	154	60	113	131	173
Entry fee		2.0%	1.7%	2.6%	1.1%	2.6%	3.1%	1.2%	2.3%	2.6%	3.5%
Exit		100	84	125	50	122	157	59	108	121	173
Exit rate		2.0%	1.7%	2.5%	1.0%	2.5%	3.2%	1.2%	2.2%	2.4%	3.5%

*Note.* Obtained from own elaboration of the Promperu database, 2009-2019.

**Figure 2.**
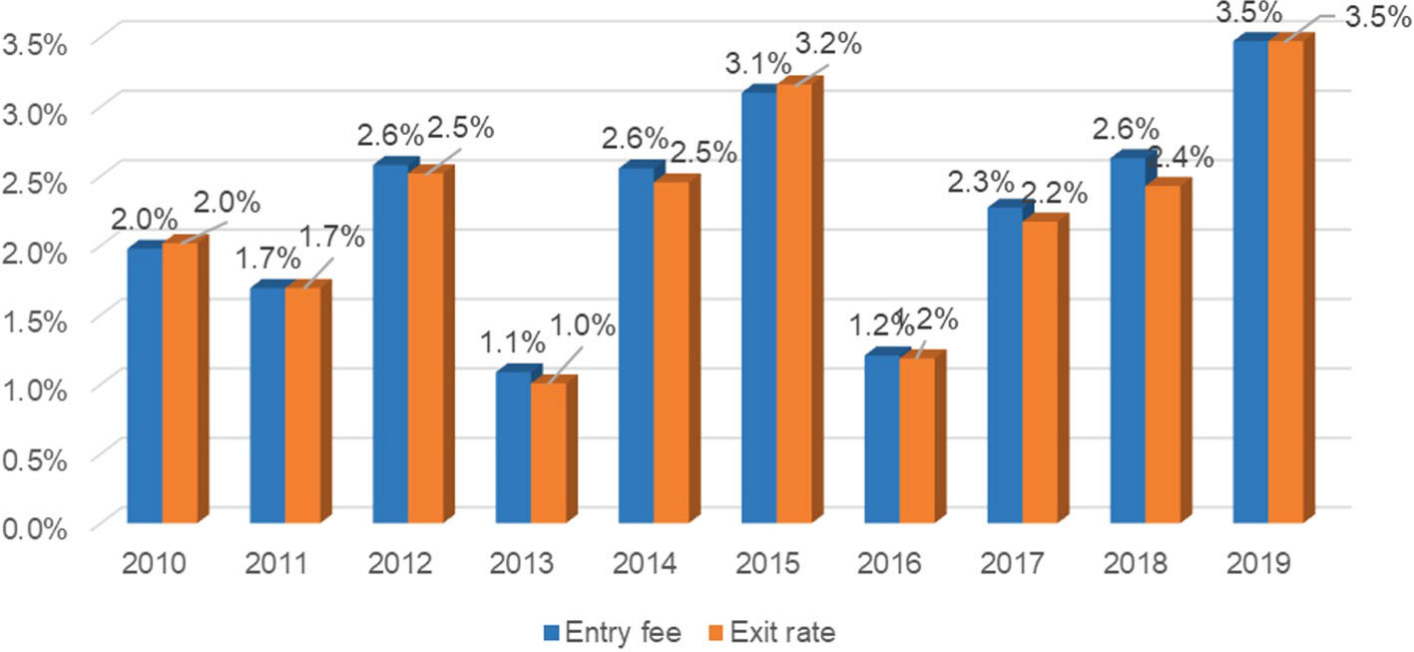
Market entry and exit rate of Peru's non-traditional agricultural sector, 2009-2019. *Note.* Obtained from own elaboration of the Promperu database, 2009-2019.

Figure 2 shows the dynamism of the entry rate, which on average reached a growth of 2.3% and the exit rate reached an average of 2.2% in the period 2009-2019, considering a relatively increasing trend from 2016 onwards, where the entry of new non-traditional agricultural companies is greater than the exit of agricultural exporting companies in Peru, after the fluctuations reflected in the period 2010-2015.

Table 3 shows that the risk factor for the survival of exporting companies in Peru’s non-traditional agricultural sector in year 1 is 0.89, which indicates that 89% of exporting companies do not survive in the export business. In year 2, 0.75 of the total number of companies survive, and so on, until year 6, when 0.33 of the total only survive to year 6.

**Table 3.** Risk factor and survival of exporting agricultural firms.

Weather	Events	Ind. risk	F. risk	F. survival
0	0	19	0	1,00
1	2	19	0,89	0,89
2	3	12	0,75	0,67
3	1	6	0,83	0,56
4	1	5	0,8	0,45
6	2	3	0,33	0,15

*Note.* Obtained from own elaboration of the Promperu database, 2009-2019.

**Figure 3.**
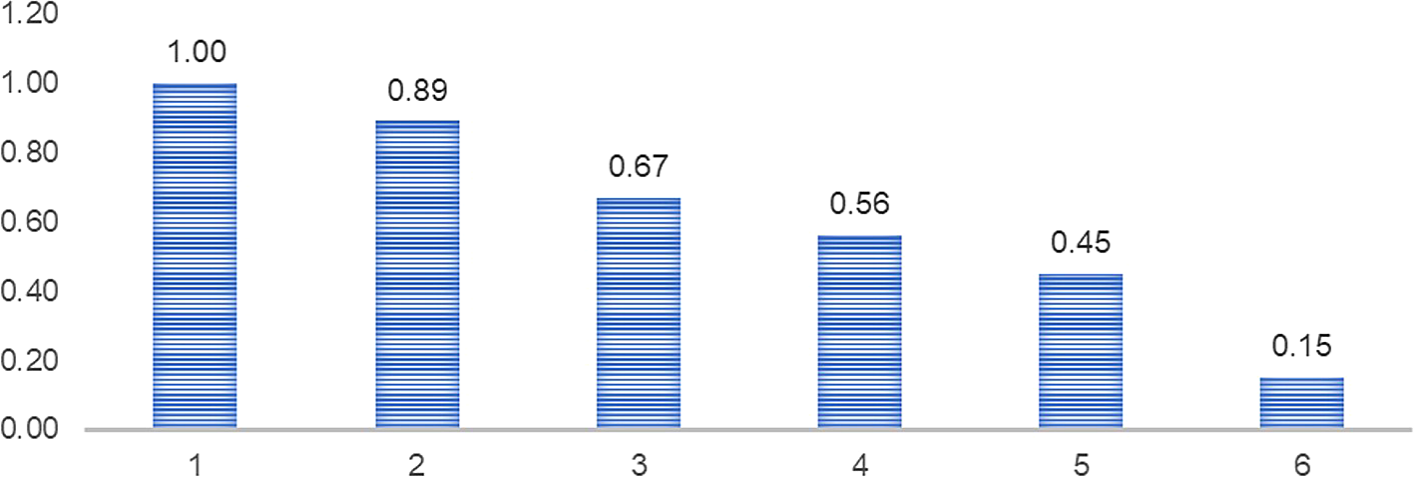
Risk factor and survival of exporting agricultural firms. *Note.* Obtained from own elaboration of the Promperu database, 2009-2019.

Figure 3 shows that the survival factor of exporting companies in Peru’s non-traditional agricultural sector, which indicates that after the first year, 11% of Peru’s exporting companies “die” or leave the market; in the third year, 33% of those that were in the first year leave the market; in the fourth year, 55% leave and so on; and by the sixth year, only 15% of those that entered the international market in the first year as exporting companies survive or remain in the international market.

In Table 4 it was observed in the analysis of the Kaplan Meier estimator that exporting companies in the non-traditional agricultural sector of less than 10 thousand dollars in FOB value have a survival time of 5 years; while exporting companies in the non-traditional agricultural sector of more than 10 thousand dollars in FOB value have a survival time of 7 years; survival being higher in those companies whose value is greater than 10 thousand dollars, being significant at 95% confidence.

**Table 4.** Means and medians for survival time.

Volume	Media ^a^				Medium			
	Estimate	Error	95% confidence interval		Estimate	Desv. Error	95% confidence interval	
			Lower limit	Upper limit			Lower limit	Upper limit
Less than $10,000	5,294	0.352	4,604	5,984	5,000	0.558	3,907	6,093
More than $10,000	6,805	0.076	6,656	6,954	7,000	0.201	6,607	7,393
Global	6,708	0.073	6,564	6,851	7,000	0.199	6,610	7,390


^a^ The estimate is limited to the longest survival time, if censored.

*Note.* Obtained from own elaboration of the Promperu database, 2009-2019.

The test of the ranks in Table 5 showed that the null hypothesis was rejected (Sig = 0.000<0.05), showing that the survival curves of exporting agricultural enterprises below 10 thousand dollars are different from those of exporting agricultural enterprises above 10 thousand dollars.

**Table 5.** Global comparisons.

	Chi-square	gl	Sig.
Log Rank (Mantel-Cox)	24,493	1	0.000

*Note.* Obtained from own elaboration of the Promperu database, 2009-2019.

Ho: Survival curves of exporting farm firms below $10,000 are equal to exporting farm firms above $10,000.

Ha: The survival curves of exporting farm firms below $10,000 are different from those of exporting farm firms above $10,000.

**Figure 4.**
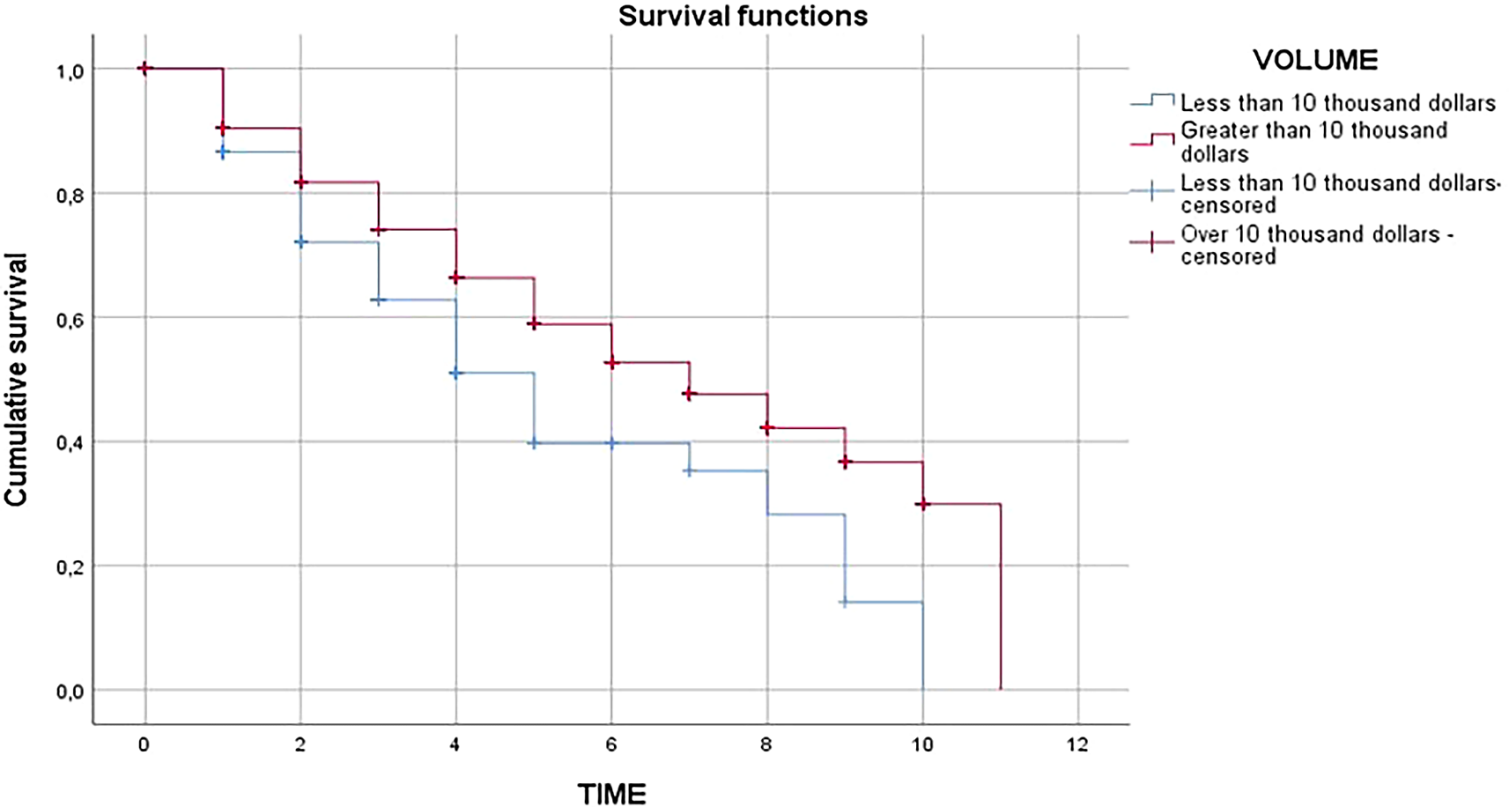
Survival functions of exporting firms in the Peruvian non-traditional agricultural sector, 2009-2019. *Note.* Obtained from own elaboration of the Promperu database, 2009-2019.

**Figure 5.**
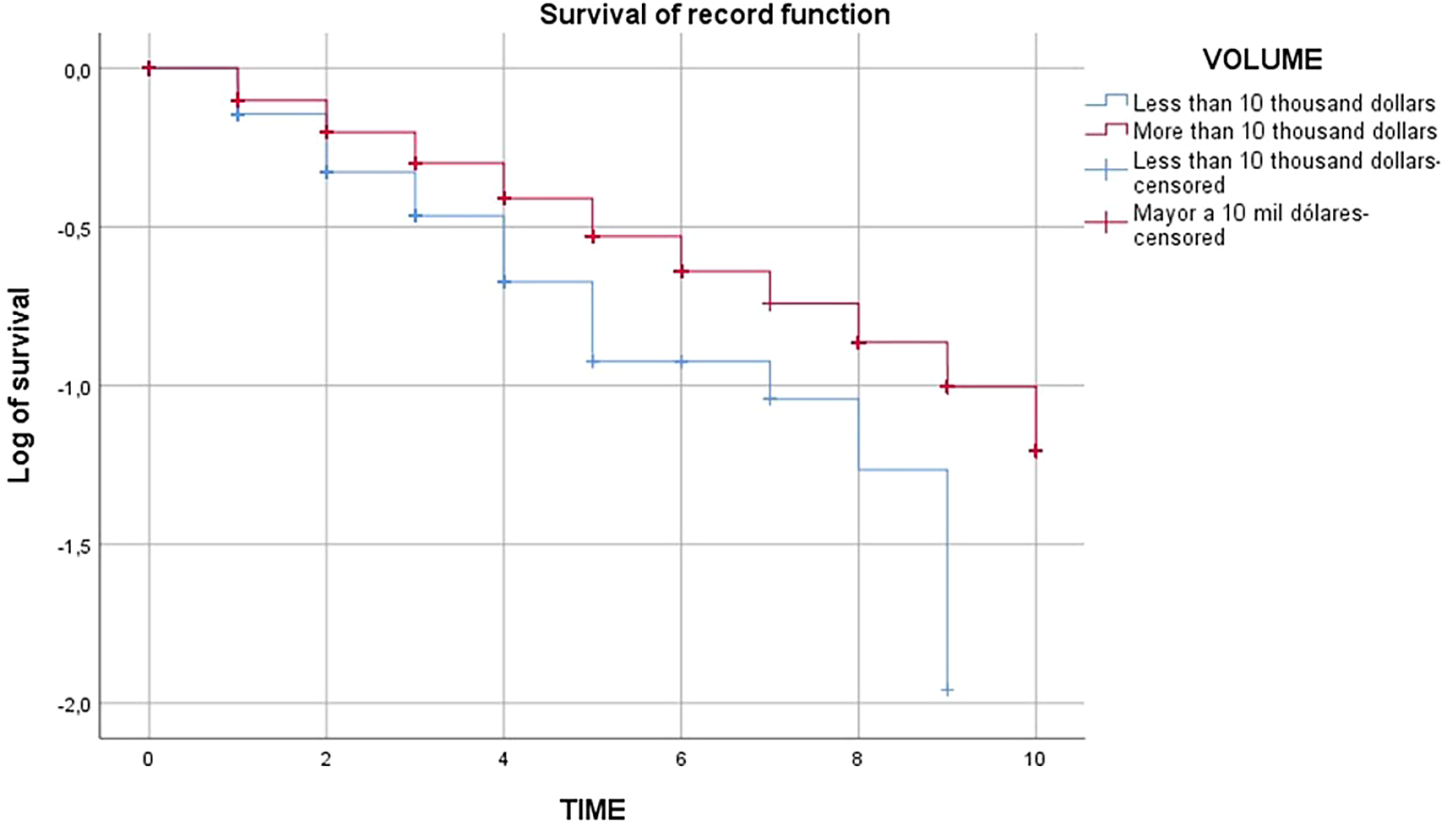
Survival functions of the register of exporting companies in Peru's non-traditional agricultural sector, 2009-2019. *Note.* Obtained from own elaboration of the Promperu database, 2009-2019.

In the contribution of Figures 4 and 5 it was shown that the survival of agricultural exporting companies larger than 10 thousand dollars is higher than agricultural exporting companies smaller than 10 thousand dollars as the red line is higher than the blue line, due to the fact that companies with higher export value would generate economies of scale and would have greater experience in the market, as well as the development of internal capacities to achieve expansion into new markets that allow them to have a greater chance of survival.

**Figure 6.**
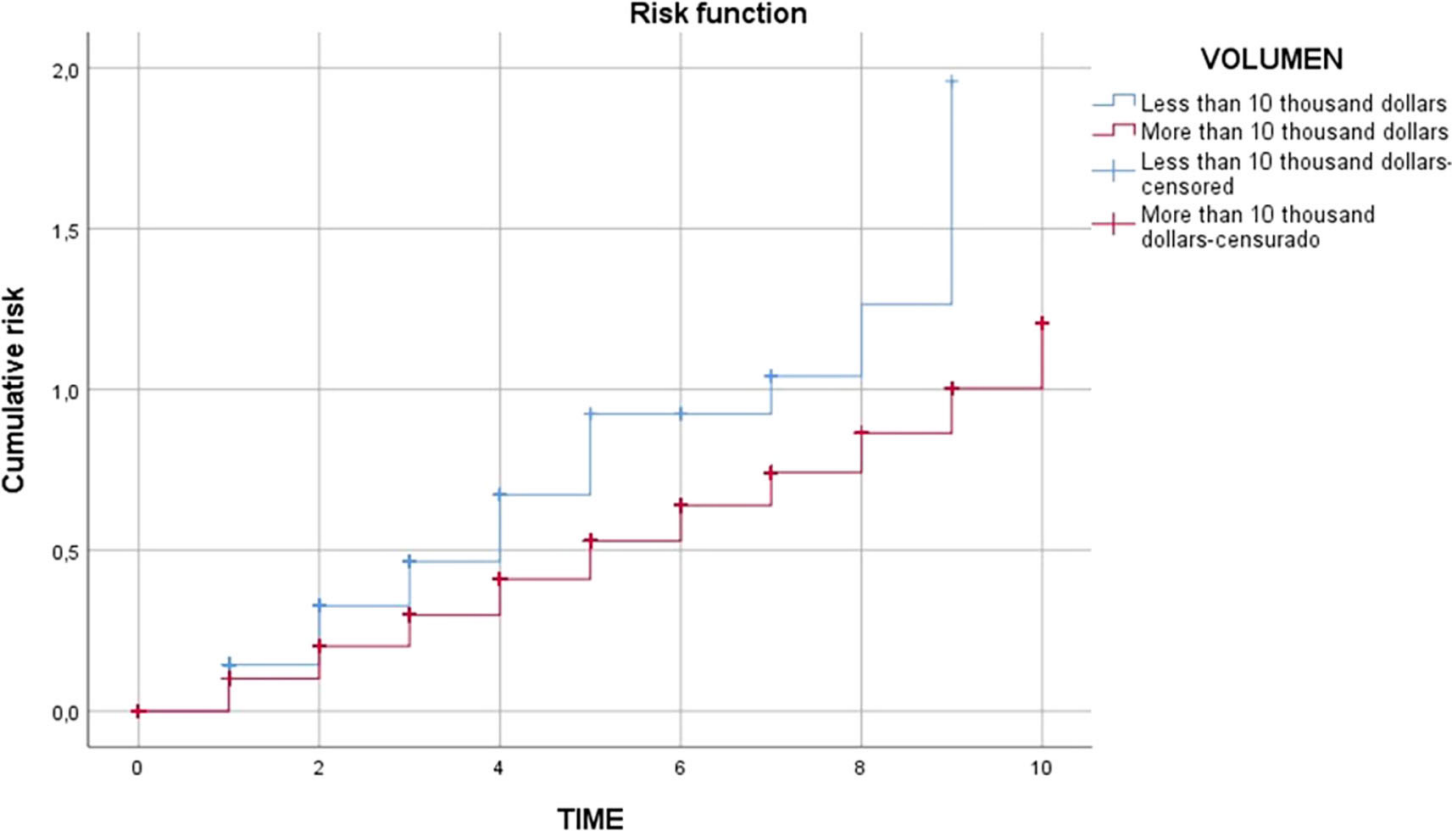
Risk function of exporting companies in Peru's non-traditional agricultural sector, 2009-2019. *Note.* Obtained from own elaboration of the Promperu database, 2009-2019.

Figure 6 shows that at the beginning of the exporting agricultural companies, both of greater and less than 10 thousand dollars in terms of FOB value, none of them are likely to leave the market; but as time progresses, the probability of leaving the market is greater for those exporting agricultural companies with an FOB value of less than 10 thousand dollars due to the limitations of both internal and external factors that do not allow them to expand into new markets, nor do they have the necessary capital to continue expanding the market and internal capacities are limited, mainly in business management, which means that the probability of the risk of leaving the market is greater.

## 6. Discussion

In relation to the general objective of carrying out an analysis of the survival of agricultural export companies in Peru, 2009-2019, it was found that 89% of them survive only one year, while in the second year only 75% survive and in the sixth year only 33% survive. In this sense, it is in agreement with what is expressed by Pantaleón et al. (2021) where avocado exporting companies reached an entry rate of 33% in 2013 and 34% by 2020 and with an exit rate that reached 32% in 2013 and 29% by 2020 and showed that the survival factor is decreasing, given that as more time passes there is less chance of survival and only the survival factor involves that in the second year 52% of companies tend to survive and in the ninth year only 6%.

Thus, the theoretical contribution of strategies for the survival of exporting companies in the business environment allows identifying that in order to survive in a business environment that is constantly changing, exporting companies manage to make use of several strategies that contribute to the adaptation of change in the global market ( Vásquez et al., 2017). Therefore, understanding these factors is essential to develop strategies that not only allow exporting companies to survive, but also to thrive in a constantly changing environment; in this way, Peruvian agro-exporting companies have identified that there are both internal and external factors that show the obstacles that limit the expansion of companies in international markets; the internal factors being those related to management focus and commitment, knowledge and experience, as well as functional and marketing information, while the external factors are those related to the destination and origin market.

In relation to the first specific objective of describing the entry and exit of agricultural exporting companies in Peru from the international market, it was found that the entry rate reached an average growth of 2.3% and the exit rate reached an average of 2.2% in the period 2009-2019, and considering a relatively increasing trend from 2016 onwards, where the entry of new non-traditional agricultural companies is greater than the exit of agricultural exporting companies in Peru. In this sense, it is in line with what is stated by Pantaleón et al. (2021) where avocado exporting companies reached an entry rate of 33% in 2013 and 34% by 2020, and with an exit rate that reached 32% in 2013 and 29% by 2020, showing that the survival factor is decreasing. They are also related to Casillas et al. (2020) the results found evidence the importance of market entry and the short-term effect, being the age of entry the one considered to be significant in explaining the volume and intensity of exports, being that exporting companies develop a gradual process in acquiring the learning of internationalisation.

In this context, from the contribution of the Conventional Trade Theory (CTT), which starts from the origin of trade by the causes of differences in technology or the aspect of preference, where trade starts by considering comparative advantages and having a preponderant role that contributes to the creation of employment and economic growth, which are essential indicators for the commercial exchange that exists between one country and another ( Fassio, 2018; Islam & Fatema, 2023; Kryeziu et al., 2024).

In relation to the second specific objective of describing the survival rate of agricultural exporting companies in Peru, it was found that the survival factor of exporting companies in the non-traditional agricultural sector in Peru is that after the first year, 11% of exporting companies in Peru “die” or leave the market; in the third year, 33% of those that were in the first year leave the market; in the fourth year, 55% leave and so on; and by the sixth year, only 15% of those that entered the international market in the first year as exporting companies survive or remain in the international market. In this sense, he agrees with Leurcharusmee et al. (2022) which finds that the survival of MSEs depends on the location, the number of employees and the adjustment of the business model, as well as the operation with social distancing and online marketing; evidencing that the trajectories of probability of survival finds that the relevant factors are those related to commercial factors such as location, total assets, type of business and commercial operation that determine the probability of survival of MSEs. Likewise Reyes (2021) based on the Logit methodology, found that the internal factors that determine the probability of an exporting company in the agricultural sector to be sustainable include the explanatory variables of access to credit, product diversification and the experience of the entrepreneurs.

From the economic contribution De Gregorio (2012) considers that exports are a boost to economic growth as they contribute to the generation of value through the trade balance for the generation of foreign currency and contribute to the gross domestic product and the diversification of the economy to maintain its competitiveness and the ability to adapt to global markets is essential for the survival of exporting companies.

In relation to the third specific objective of carrying out a comparative analysis of the survival time of agricultural exporting companies in Peru, it showed that the survival of agricultural exporting companies of more than 10,000 dollars is higher than that of agricultural exporting companies of less than 10,000 dollars as the red line is higher than the blue line, due to the fact that companies with greater export value would generate economies of scale and would have greater experience in the market, as well as the development of internal capacities to achieve expansion into new markets that would allow them to have a greater chance of survival. In this sense, it agrees with Safari and Saleh (2020) involves that in emerging countries internal and external factors have been identified on the aspect of innovation strategy, export marketing and business strategy that involves improving the export process and motivating companies to export by applying effective standards, simplifying processes, increasing incentives and other strategic initiatives to achieve export to regional and global markets.

Since the contribution of the Kaplan-Meier method is of great importance in the field of statistics and medical research, it provides a robust and detailed way of analysing survival data. This method is especially useful because it can handle censored data, i.e. data where complete information on the survival time of all individuals in the study is not available.

The Kaplan-Meier method is an essential statistical tool that allows researchers to effectively handle incomplete data, provide accurate estimates of survival, and visualise differences between study groups, facilitating informed decision-making and improved interventions and treatments ( Gémar et al., 2016).

## 7. Conclusions

The survival of exporting companies in the non-traditional agricultural sector is critical, where 89% of them survive only one year, while in the second year only 75% survive and in the sixth year only 33% survive.

Peru’s non-traditional agricultural sector exports in FOB value have had an average annual growth of 12% in FOB value and 9% in volume exported; the main agricultural agro-export products in Peru are asparagus, grapes, coffee, avocado, blueberries, mango and quinoa; these products not only generated significant income for the country, but also promoted the development of agriculture and related infrastructure, benefiting numerous rural communities.

The entry rate of new agro-exporting agricultural companies reached an average growth rate of 2.3% and the exit rate reached an average of 2.2% in the period 2009-2019; with a relatively increasing trend from 2016 onwards where the entry of new non-traditional agricultural companies is higher than the exit of agricultural exporting companies in Peru, after the fluctuations reflected in the period 2010-2015.

The comparative analysis of the survival time of agricultural exporting companies in Peru showed that those companies with an FOB value of less than 10,000 dollars had a survival time of 5 years, while companies with an FOB value of more than 10,000 dollars had a survival time of 7 years, with a higher survival time for those companies with a value of more than 10,000 dollars.

The survival rate of exporting companies in the non-traditional agricultural sector in Peru, which was carried out using the Kaplan Meier index, considers that 11% of the companies die in the first year of activity, in the second year they are no longer in activity, in the third year only 33% remain, in the fourth year 55% and in the sixth year only 15%.

### Ethics and consent

Ethics and Consent were not required for the performed study.

## Data Availability

Zenodo: Analysis of the survival of agricultural exporting firms in Peru, 2009-2019. Version 1. https://doi.org/10.5281/zenodo.14002405. ( Morán et al., 2024). The project contains the following underlying data:
• Kappla-Meier database (Result of the survival analysis of agricultural exporting firms, using the Kaplan Meier estimator over the estimated time period from 2009 to 2019). • Data SPSS.sav (Data analysis was conducted using IBM SPSS Statistics 27 software) Data are available under the terms of the Creative Commons Zero v1.0 Universal (CC0 1.0) Zenodo: Analysis of the survival of agricultural exporting firms in Peru, 2009-2019. Version 1. https://doi.org/10.5281/zenodo.14002405 ( Morán et al., 2024). This project contains the following extended data:
• Supplementary Figure 1. (Net balance of exporting firms in Peru’s non-traditional agricultural sector, 2009-2019). • Supplementary Figure 2. (Market entry and exit rate of Peru’s non-traditional agricultural sector, 2009-2019). • Supplementary Figure 3. (Risk factor and survival of exporting agricultural firms). • Supplementary Figure 4. (Survival functions of exporting firms in the Peruvian non-traditional agricultural sector, 2009-2019). • Supplementary Figure 5. (Survival functions of the register of exporting companies in Peru’s non-traditional agricultural sector, 2009-2019). • Supplementary Figure 6. (Risk function of exporting companies in Peru’s non-traditional agricultural sector, 2009-2019). Data are available under the terms of the Creative Commons Zero v1.0 Universal (CC0 1.0) Zenodo: STROBE Statement - checklist in reports of observational studies for ‘Analysis of the survival of agricultural exporting firms in Peru, 2009-2019’. Version 1. https://doi.org/10.5281/zenodo.14002405 ( Morán et al., 2024). Data are available under the terms of the Creative Commons Zero v1.0 Universal (CC0 1.0)
