# Supplementary material for: Analysis of the survival of agricultural exporting firms in Peru, 2009-2019
Source: F1000Res. 2024 Nov 27;13:1437. [Version 1] doi: 10.12688/f1000research.158554.1 (PMC12572774; doi:10.12688/f1000research.158554.1)
Supplement: Peer review history

# Reviewer report: Reviewer response for version 1

**DOI:** 10.5256/f1000research.174163.r420636

**Published:** 2025-10-31

**Authors:** Luis Eduardo Solis Granda

**Affiliations:** 1 Universidad Estatal de Milagro, Milagro, Ecuador

This study analyzes the survival of non-traditional agricultural exporting companies in Peru (2009-2019) using Kaplan-Meier survival analysis. The authors obtain data from Promperu (official export promotion agency) covering 4,961 companies, calculate market entry/exit rates (2.3% and 2.2% respectively), and compare survival between firms with exports <$10,000 vs. >$10,000 FOB value. Main findings: 89% survive year one, 75% survive year two, 33% survive year six. The authors conclude that exporter survival is "critical" and that larger export volumes correlate with longer operational duration.

 The work is acceptable as a descriptive study but limited in explanatory depth. It competently addresses "what survival rates are" but not "why firms exit." The methodology (Kaplan-Meier) is appropriate, and data are from reliable source. However, critical ambiguities in operational definitions (what is "exit"?) and the unexplained reduction to n=19 undermine confidence in reported estimates. The paper would become a solid contribution with: (1) clarification of exit definition with sensitivity analysis, (2) explanation of n=19 reduction, (3) expansion to multivariable Cox regression to identify predictors of survival, and (4) elimination of redundant presentation. In current form, it documents survival patterns but offers limited new insights beyond Pantaleón et al. (2021) and provides no guidance for policy intervention.

Is the work clearly and accurately presented and does it cite the current literature?

Partly

If applicable, is the statistical analysis and its interpretation appropriate?

Yes

Are all the source data underlying the results available to ensure full reproducibility?

Partly

Is the study design appropriate and is the work technically sound?

Partly

Are the conclusions drawn adequately supported by the results?

Partly

Are sufficient details of methods and analysis provided to allow replication by others?

Partly

Reviewer Expertise:

Organizational Behavior, Operations Management, Corporate Social Responsibility, and Business Management

I confirm that I have read this submission and believe that I have an appropriate level of expertise to confirm that it is of an acceptable scientific standard, however I have significant reservations, as outlined above.

# Reviewer report: Reviewer response for version 1

**DOI:** 10.5256/f1000research.174163.r420634

**Published:** 2025-10-29

**Authors:** Cristian Guzmán Cofre

**Affiliations:** 1 Católica de la Santísima Concepción, Concepción, Chile

The literature review is very general with regard to exporting companies but lacks precision in analyzing specific issues related to exporting firms in the agricultural sector, which is the subject of study. 

 There is no discussion of the possibility that the companies that are exporting are those that already have a position in the domestic market and decide to export out of a need for growth or as part of their strategy. This is because the position in the domestic market may also be a determinant of the volume of exports of firms, which would support a longer duration in the international market. Now, if this analysis was not done, it would be good to state this in the limitations of the study, since there is a discussion in the literature regarding the existence of exports by companies as a development strategy or as a result of an already strong position in the domestic market. 

 In the unit of analysis, the methodology looks at exporting firms with a threshold of US$10,000, which is understood to come from the analysis of the data. But to complement this, is there not a measure that classifies exporting companies by international sales bracket, or some international classification measure that allows results to be compared in other studies? Because it may be that survival is also determined by the size of the firm, which correlates with sales volume and the size of companies by number of employees. If no such classification exists or is not relevant to the study, I suggest that this be made explicit in the document.

Is the work clearly and accurately presented and does it cite the current literature?

Partly

If applicable, is the statistical analysis and its interpretation appropriate?

Yes

Are all the source data underlying the results available to ensure full reproducibility?

Yes

Is the study design appropriate and is the work technically sound?

Yes

Are the conclusions drawn adequately supported by the results?

Yes

Are sufficient details of methods and analysis provided to allow replication by others?

Partly

Reviewer Expertise:

innovation strategies, economic decisions of the firm, innovation activities, eco-innovation

I confirm that I have read this submission and believe that I have an appropriate level of expertise to confirm that it is of an acceptable scientific standard.

# Reviewer report: Reviewer response for version 1

**DOI:** 10.5256/f1000research.174163.r402450

**Published:** 2025-09-25

**Authors:** Victor Hugo Puican Rodríguez

**Affiliations:** 1 Universidad Nacional Intercultural Fabiola Salazar Leguia de Bagua, Bagua, Peru

Methodology: More detail on technical aspects.

 It would be advisable to clarify more precisely how censored observations are treated in the survival analysis, explaining what happens to companies that remain active at the end of the study period or that change their line of business.

 With regard to the Kaplan-Meier estimator, although its application is described, it would be preferable to adopt the standard formal notation and include a concise explanation of its practical interpretation in a corporate context. This would help readers without statistical knowledge to better understand the results.

Is the work clearly and accurately presented and does it cite the current literature?

Partly

If applicable, is the statistical analysis and its interpretation appropriate?

Yes

Are all the source data underlying the results available to ensure full reproducibility?

Partly

Is the study design appropriate and is the work technically sound?

Partly

Are the conclusions drawn adequately supported by the results?

Yes

Are sufficient details of methods and analysis provided to allow replication by others?

Partly

Reviewer Expertise:

Exportación, finanzas, economía. contabilidad, gestión.

I confirm that I have read this submission and believe that I have an appropriate level of expertise to confirm that it is of an acceptable scientific standard.
